# Epidermal to Mesenchymal Transition and Failure of EGFR-Targeted Therapy in Glioblastoma

**DOI:** 10.3390/cancers4020523

**Published:** 2012-05-08

**Authors:** Andrej Pala, Georg Karpel-Massler, Richard Eric Kast, Christian Rainer Wirtz, Marc-Eric Halatsch

**Affiliations:** 1 Department of Neurosurgery, University of Ulm School of Medicine, Steinhövelstrasse 9, Ulm D-89077, Germany; E-Mails: andrej.pala@uniklinik-ulm.de (A.P.); georg.karpel@uniklinik-ulm.de (G.K.-M.); rainer.wirtz@uniklinik-ulm.de (C.R.W.); 2 Department of Psychiatry, University of Vermont, 22 Church Street, Burlington, VT 05401, USA; E-Mails: richarderickast@gmail.com (R.E.K.)

**Keywords:** epithelial to mesenchymal transition, glioblastoma multiforme, targeted therapy, epidermal growth factor receptor, EGFRvIII, tyrosine kinase, erlotinib, gefitinib

## Abstract

Glioblastoma multiforme (GBM), the most common primary brain tumor in adults, is almost never curable with the current standard treatment consisting of surgical resection, irradiation and temozolomide. The prognosis remains poor despite undisputable advances in the understanding of this tumor’s molecular biology and pathophysiology, which unfortunately has so far failed to translate into a meaningful clinical benefit. Dysregulation and a resulting prominent pathophysiological role of the epidermal growth factor receptor (EGFR) have been identified in several different malignant tumor entities, GBM among them. The EGFR is overexpressed in about 40% of GBM cases, and half of these coexpress a mutant, constitutively activated subtype, EGFRvIII. Unfortunately, recent trials studying with therapeutic approaches targeted against the EGFR and EGFRvIII have failed to meet expectations, with only a minority of patients responding despite evidence of good *in vitro* and rodent model activity. Having potentially high relevance within this context, epithelial to mesenchymal transition (EMT) is a phenomenon associated with early stages of carcinogenesis, cancer invasion and recurrence. During EMT, epithelial cells lose many of their epithelial characteristics, prominently E-cadherin expression, and acquire properties that are typical for mesenchymal cells such as the expression of vimentin. Epithelial to mesenchymal transition has been specifically demonstrated in GBM. In this review, we summarize the evidence that EMT may precipitate GBM resistance to EGFR-targeted therapy, and may thus be among the principal factors contributing to the clinical failure of targeted therapy against EGFR and EGFRvIII.

## 1. Introduction

The epidermal growth factor receptor (EGFR) has emerged as a promising therapeutic target because it is overexpressed in a wide range of cancers and plays important roles in cell growth and survival. The EGFR is a transmembraneous glycoprotein, synonymous with HER-1, composed of an external domain that binds activating ligands such as EGF and tumor growth factor-α (TGF-α), and an intracellular tyrosine kinase (TK) domain, which on activation phosphorylates a variety of downstream effector molecules such as phospholipase Cγ, phophatidylinositol-3-kinase and mitogen-activated kinase [1,2,3,4,5,6]. Against this background, several drugs that target the EGFR have emerged, including the small molecule TK inhibitors, e.g., erlotinib and gefitinib, and a monoclonal antibody directed against the EGFR, cetuximab [2,4,7]. Unfortunately, in recent clinical trials, erlotinib improved disease control only in a minority of patients with GBM [8].

Epithelial to mesenchymal transition (EMT) refers to a process where sessile epithelial cells acquire a loss of attachment and new migratory behavior, and its presence predicts *in vitro* resistance of non-small cell lung cancer (NSCLC), pancreatic and colorectal cancer cell lines to erlotinib [1,3,9]. Similar results have been reported regarding gefitinib sensitivity in cell lines from NSCLC and squamous cell carcinoma of the head and neck (SCCHN) [1,5,7,10].

## 2. E-Cadherin and EMT

Epithelial to mesenchymal transition plays important roles in normal organ development, wound healing, housekeeping organ maintenance, and cancer progression. The process is characterized by the combined loss of sessile epithelial cells and of E-cadherin, and the gain of mesenchymal markers such as fibronectin and vimentin, and changes of biological behavior such as increased invasion and migration [6,8,9,11,12]. One of the early events in EMT is the disassembly of tight junctions, which results in the redistribution of Zonula occludens proteins, *i.e*., claudin and occludin. This process initiates disruption of the polarity complex and cytoskeleton reorganization [11]. The epithelial layer is polarized in a way that bottom and top can be defined as basal and apical. The filamentous actin cytoskeleton is apicobasally polarized and shows circumferential organization. The main intermediate filaments are represented by cytokeratins [11]. Loss of E-cadherin with consequent disassembly of adherens junctions, located adjacent to tight junctions in the basolateral surface compartments of epithelial cells, is considered a hallmark of EMT [1,2,3,4,13,14,15]. Adherens junctions build a belt-like connection at the lateral interface of epithelial cells. The transmembrane adhesion receptor E-cadherin spans the intercellular space between neighboring cells. The cytoplasmic domains of E-cadherin build complexes with β-catenin, a cytoplasmic protein that interacts with α-catenin, which anchors to the actin cytoskeleton, either directly or indirectly via the actin-binding proteins α-actinin und vinculin [13]. During EMT the adherens junctions disassemble, and the actin cytoskeleton reorganizes itself from an epithelial cortical alignment to one of mesenchymal identity. Mesenchymal cells build a diffuse network with certain fixation points adhering to their neighbor cells and with diffuse contacts to the extracellular matrix [11].

Another structure, providing additional strength at the lateral side of the epithelial cells, is the desmosome [11]. Desmosomes are similar to adherens junctions, however organized as individual patches. A decrease in the expression of desmoplakin, an obligate component of functional desmosomes that anchors intermediate filaments to desmosal plaques, and of desmosome components, has been described in EMT [11,13]. The loss of cellular polarity and the change of the cellular shape, eventually forming a spindle meshwork, enhance the separation and migration potential of the cell [11].

## 3. “Go or Grow”

Gliomas not only proliferate but also invade the surrounding brain parenchyma, paving the path to recurrence of the tumor. Using mathematical modeling, Tektonidis *et al*. have predicted the expansion speed and the invasive zone width of infiltrating glioma cells [16]. Typically, gliomas consist of two main parts, the solid and/or necrotic “core” of the tumor and its infiltrative zone. Experimental evidence suggests that the invasive cells are of a different phenotype than the core cells and have different motility and proliferation rates. There is a difference in the speed of progression in the central core and the peripheral rim. Cells located in the core are able to proliferate faster than those at the invasive edge [16]. In particular, it has been shown that there is an inverse correlation of cell motility and proliferation. Highly motile glioma cells tend to have lower proliferation rates than the less migratory cells. This concept suggests that migration and proliferation are mutually exclusive processes (“Go or Grow”). As a result, the invasive rim develops faster than the core. In case that in the core region no mitosis occurs, all cells migrate and contribute to the invasive rim. The structure of the core region is lost under these circumstances. Another aspect is to view the cell density itself as a source of signaling events that will alter cell motility and/or cell growth. This switch can regulate the balance of migration and proliferation [16].

## 4. Matrix Metalloproteases (MMPs) and TGF-β as EMT Inducers

**Figure 1:**
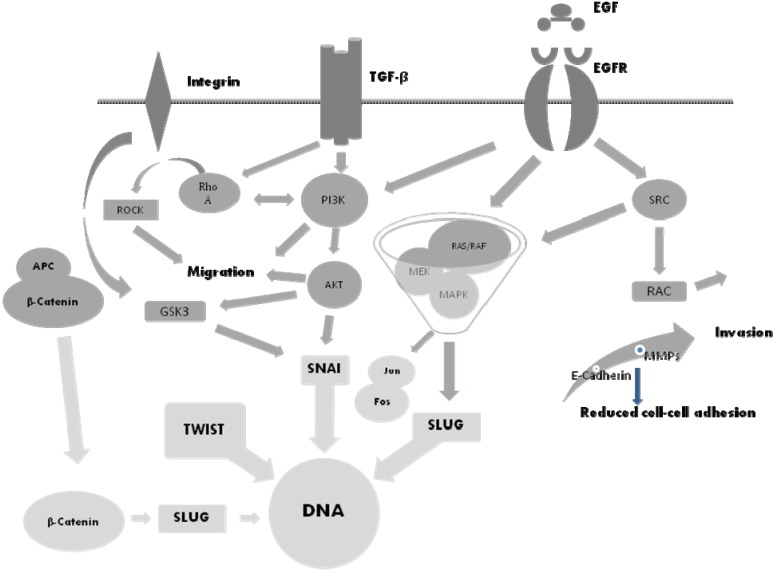
Representation of the signal transduction pathways associated with EMT. Abbreviations: TGF-β, transforming growth factor β; EGF, epidermal growth factor; FGF, fibroblast growth factor; HGF, hepatocyte growth factor; IGF, insulin-like growth factor; PI3K, phosphoinositide 3-kinase; ILK, integrin-linked kinase; APC, adenomatous polyposis coli; GSK 3, glycogen synthase kinase; RTK, receptor tyrosine kinase.

Tumor cells express MMPs that dissolve the extracellular matrix and thereby promote invasion, migration and metastasis [11]. In this context, TGF-β is one of the most potent EMT inducers present in the tumor environment and assumes an important role [13,17]. Transforming growth factor-β, a protein molecule, is able to induce apoptosis in numerous cell types through the Smad pathway. In addition, TGF-β acts through other non-Smad pathways, including the MMP cascade, to regulate gene expression associated with malignant transformation. Metalloproteinases belong to a large family of more than 28 proteins, zinc-dependent endopeptidases, all of which attack extracellular matrix proteins. A potential connection between EMT and MMPs has been suggested in recent studies, particularly for MMP-2 and MMP-9 [13,17,18]. Transforming growth factor-β1 takes part in the initiation of EMT (Figure 1), which is mediated further by SNAI-1 and SLUG which in turn regulate MMP-2 and MMP-9. Matrixmetalloprotease-2 and MMP-9 have a similar molecular structure, except that the precursor form of MMP-2 requires cleavage of the N-terminal prodomain to become active. This is a prerequisite for SNAI regulation and can be performed by a number of processes, for instance via nuclear factor κB (NF-κB). In addition to the above, TGF-β is a cytokine that is released by glioma cells in large quantities *in vitro* and *in vivo* and has been implicated in the malignant progression of glial tumors and the immune dysfunction in patients with GBM by promoting tumor-angiogenesis, enhancing invasion, migration and inhibiting T cell-mediated immune responses. Thus, EMT is not the sole consequence of TGF-β-mediated stimulation [13,17].

## 5. The Molecular Basis of EMT and Potential Therapeutic Targets

The following growth factors are known to induce EMT: members of the EGF family, fibroblast growth factor, insulin-like growth factor and hepatocyte growth factor. The key transcription factors regulating EMT are SNAI-1/2, zinc finger E-box-1/2 (ZEB-1/2) and TWIST with WNT/β-catenin [11,14,18,19,20,21,22,23]. The SNAI family members (SNAI-1-3) are closely related transcriptional repressors. Their *C*-terminal regions contain zinc-finger domains which attach to specific DNA sequences, and their *N*-terminal regions contain a SNAG domain which is critical for their activity [18,20]. SNAI-1 is expressed during mesoderm formation, gastrulation and neural crest development. Overexpression of SNAI-1 is alone sufficient to induce EMT *in vitro* [13,17,18,20]. Moreover, it has been shown to correlate closely with invasiveness and recurrence of breast and colon cancer [11].

TWIST-1 is a helix-loop-helix protein that represses the epithelial factor E-cadherin and activates the mesenchymal marker *N*-cadherin, a hallmark feature of carcinoma EMT termed the “cadherine switch” [8,20]. It has been demonstrated that in gliomas TWIST-1 promotes cellular invasion through activation of mesenchymal and molecular changes [14], avoiding the typical pathway by using the cadherine switch. Specific inhibition of TWIST expression resulted in a reduction of glioma cell invasion *in vitro* [14]. Thus, targeting TWIST-1-mediated mesenchymal changes represents a potential treatment option to inhibit invasion of glioblastoma cells.

Other essential transcription factors in EMT are ZEB-1 (also termed delta-crystalline enhancer binding factor-1) and ZEB-2 (as mentioned above) which represent critical regulators of TGF-β-mediated signaling through physical interactions with Smad proteins to recruit coactivators and corepressors. The ZEB proteins are implicated in EMT in several tumor types. The WNT/β-catenin pathway induces cell migration which is necessary for pattern formation and differentiation during embryonic development and tumor progression. Moreover, WNT is also considered an important regulator that promotes cellular invasiveness through regulation of EMT in many neoplastic entities. WNT signaling is frequently activated in colorectal cancers, melanomas, breast cancers, and gliomas due to loss of adenomatous polyposis coli (APC) function and gain of β-catenin activity. It might be a potential driver that leads to acquisition of both stemness and invasiveness of GBM cells [19]. Zinc finger E-box-1 regulates E-cadherin transcription by binding of the two zinc finger domains to two E-boxes located in the E-cadherin promoter region [7]. Haddad *et al*. have shown that the resistance to erlotinib may be linked to EMT driven by ZEB-1 in human SCCHN cell lines [7]. Cells sensitive to erlotinib express high levels of E-cadherin and low levels of ZEB-1. On the other hand, the drug-resistant cells express high levels of ZEB-1, knockdown of which restored sensitivity to erlotinib and enabled to revert the EMT phenotype in mesenchymal cell lines [7].

Signal transducer and activator of transcription-5 (STAT 5) represents a transcription factor that is responsible for the regulation of many genes when stimulated by a variety of growth factors. Signal transducer and activator of transcription-5 dimers anchor to specific DNA promoter regions and regulate cellular growth, migration and motility [24]. Dysregulation of STAT-5 is present in many tumors including prostate cancer, breast cancer, nasopharyngeal carcinoma and SCCHN of the head and neck. It has been shown that activation of STAT-5 in SCCHN leads to decreased growth inhibition by erlotinib and decreased apoptosis induction by cisplatin, and thereby to a diminished clinical effect [24]. The constant activation of STAT-5 reduced expression of E-cadherin and increased expression of vimentin, consistent with induction of EMT [24].

The infiltrative property of GBM cells is one of the major causes of tumor recurrence and patient lethality, because GBM cells that infiltrate the parenchyma are difficult to eradicate with local therapeutic modalities, being extremely resistant to various anticancer therapies. The fact that the mesenchymal change in GBM is associated with a more aggressive clinical phenotype suggests that mechanisms that promote EMT in carcinoma may be valuable therapeutic targets in the future treatment of GBM as well [1,2,3,4,16].

## 6. The SNAI Family

Recent studies have shown that EMT-related genes are involved in gliomagenesis. The expression level of E-cadherin, which is a target gene of SNAI, is lower in high-grade gliomas than in low-grade gliomas [15]. Brain tissue shows a higher expression of E-cadherin than malignant astrocytomas. SMAD-interacting protein-1, which is one of the critical regulators of EMT, is expressed in glioma cell lines and is known to regulate invasion, migration and clonogenicity of glioma cells [21]. Yang *et al*. showed that SNAI-2 is overexpressed in GBM and promotes the growth and invasion of this tumor [22]. Han *et al*. performed SNAI-1 expression knockdown using small interfering RNA in glioblastoma cell lines and confirmed that SNAI-1 is involved in the proliferation and migration of glioblastoma cells [18]. The knockdown of SNAI-1 led to decreased cellular invasion and migration. The underlying biological effects of SNAI-1 have been reported for many other cancers. SNAI-1 is a potent regulator of E-cadherin and induces MMP-2 expression which eventually promotes invasion of carcinoma cells.

Chi *et al*. have reported a case of clinical improvement after treatment of a MET-amplified recurrent glioblastoma with crizotinib [25]. Crizotinib is an anaplastic lymphoma kinase (ALK) inhibitor, approved for the treatment of NSCLC. The compound also inhibits the MET/hepatocyte growth factor receptor tyrosine kinase. Various mechanisms, such as gene mutation, amplification or rearrangement are responsible for the malignant transformation of gliomas [25,26]. The protooncogene MET (c-met) is amplified in human gliomas and causes increased activity of its downstream targets [26], resulting in enhanced proliferation, invasion, migration, angiogenesis and survival of malignant gliomas [25]. Moreover, MET amplification is associated with a worse clinical outcome. Some gliomas may be dependent on MET amplification and therefore respond to MET inhibitors [25,26].

## 7. Conclusions

Epithelial to mesenchymal transition is a phenomenon during which epithelial cells lose many of their epithelial characteristics and acquire markers of mesenchymal cells. This transformation contributes to the highly invasive nature of gliomas. Invasive GBM cells infiltrate the brain parenchyma and escape surgical resection and other local therapeutic modalities, and are considered a principle reason for tumor recurrence. Glioblastoma patients have a poor prognosis with a median survival of shortly over one year [8]. Targeted therapy against EFGR and EGFRvIII has shown no survival benefit compared to standard therapy, and EMT must be considered an important mechanism contributing to failure of this approach. In its complexity, EMT encompasses many pathways that promote the continuous acquisition of malignant biological features by glioma cells. A better understanding of the molecular basis of EMT may stimulate the identification of new molecular targets for rationally designed combination therapies as well as of patient subgroups who may benefit from EGFR inhibition.
